# Estudio transversal de las parasitosis intestinales en poblaciones infantiles de Argentina

**DOI:** 10.26633/RPSP.2017.24

**Published:** 2017-03-06

**Authors:** Graciela Teresa Navone, María Lorena Zonta, Paola Cociancic, Mariela Garraza, María Inés Gamboa, Luis Alberto Giambelluca, Silvia Dahinten, Evelia Edith Oyhenart

**Affiliations:** 1 Centro de Estudios Parasitológicos y de Vectores Universidad Nacional de La Plata, Consejo Nacional de Investigaciones Científicas y Técnicas La Plata Argentina Centro de Estudios Parasitológicos y de Vectores, Universidad Nacional de La Plata, Consejo Nacional de Investigaciones Científicas y Técnicas, La Plata, Argentina.; 2 Instituto de Genética Veterinaria Ing. Fernando Noel Dulout Facultad de Ciencias Veterinarias, Universidad Nacional de La Plata, Consejo Nacional de Investigaciones Científicas y Técnicas La Plata Argentina Instituto de Genética Veterinaria Ing. Fernando Noel Dulout, Facultad de Ciencias Veterinarias, Universidad Nacional de La Plata, Consejo Nacional de Investigaciones Científicas y Técnicas, La Plata, Argentina.; 3 Facultad de Ciencias Veterinarias Universidad Nacional de La Plata La Plata Argentina Facultad de Ciencias Veterinarias, Universidad Nacional de La Plata, La Plata, Argentina.; 4 Centro Nacional Patagónico Consejo Nacional de Investigaciones Científicas y Técnicas Puerto Madryn, Chubut Argentina Centro Nacional Patagónico, Consejo Nacional de Investigaciones Científicas y Técnicas, Puerto Madryn, Chubut, Argentina.

**Keywords:** Parasitosis intestinales, niños, Argentina, Intestinal Diseases, parasitic, child, Argentina

## Abstract

**Objetivo.:**

*Determinar la distribución de las enteroparasitosis en niños de nueve provincias representativas del mosaico de ambientes contrastantes de Argentina*.

**Métodos.:**

*Estudio descriptivo, observacional y transversal en niños preescolares (de 5 años o menos) y escolares (de 6 a 14 años) de las provincias de Buenos Aires (muestra tomada entre 2005 y 2013), Chubut (2010-2013), Corrientes (2012), Entre Ríos (2010-2012), Formosa (2014), La Pampa (2006), Mendoza (2008-2011), Misiones (2005-2008 y 2013) y Salta (2012-2013). Se procesaron muestras seriadas, fecales y de escobillado anal, mediante técnicas de concentración. Los resultados se analizaron por sexo, intervalo de edad y provincia. Se calcularon la frecuencia de parasitosis (monoparasitosis y parasitosis múltiple), la riqueza de especies y el coeficiente de similitud de Sørensen*.

**Resultados.:**

*Misiones presentó la mayor frecuencia de niños parasitados y Chubut la menor (82,0% vs. 38,4%; p < 0,01). El número de especies fue mayor en Misiones y Buenos Aires y menor en Chubut y La Pampa. Los varones estuvieron más parasitados que las mujeres solo en Buenos Aires. Las mayores frecuencias se encontraron en los preescolares de Buenos Aires y los escolares de Mendoza y Misiones (p < 0,05). La monoparasitosis fue más frecuente en Chubut (67,9%) y las parasitosis múltiples en Formosa (69,2%). Las especies más frecuentes en la mayoría de las provincias fueron* Blastocystis *sp. y* Enterobius vermicularis. *De los geohelmintos, Misiones presentó la mayor frecuencia (23,3%) y Mendoza la menor (0,6%); no se hallaron en Chubut, La Pampa y Salta. Buenos Aires, Formosa y Misiones presentaron una composición de especies similar, al igual que Chubut y La Pampa*.

**Conclusiones.:**

*Las frecuencias de parasitosis en Argentina responden al complejo mosaico de variabilidad climática y socioeconómica del país y revelan una tendencia descendente de norte a sur y de este a oeste*.

## Abstract (en)

**Objective.:**

Determine the distribution of intestinal parasitosis in children in nine provinces representative of Argentina’s mosaic of contrasting environments.

**Methods.:**

Descriptive, observational, cross-sectional study of preschool children (5 years old or under) and school-age children (6-14 years) in the provinces of Buenos Aires (sample taken between 2005 and 2013), Chubut (2010-2013), Corrientes (2012), Entre Ríos (2010-2012), Formosa (2014), La Pampa (2006), Mendoza (2008-2011), Misiones (2005-2008 and 2013), and Salta (2012-2013). Serial samples, fecal samples, and anal swabs were processed using concentration techniques. The results were analyzed by sex, age interval, and province. Frequency of parasitosis (monoparasitosis and multiple parasitoses), wealth of species, and Sørensen similarity coefficient were calculated.

**Results.:**

Misiones presented the highest frequency of parasitized children and Chubut the lowest (82.0% *vs* 38.4%; *p* < 0.01). The number of species was greatest in Misiones and Buenos Aires and lowest in Chubut and La Pampa. Men were parasitized more than women only in Buenos Aires. The highest frequencies were found in preschool children in Buenos Aires and schoolchildren in Mendoza and Misiones (*p* < 0.05). Monoparasitosis was most frequent in Chubut (67.9%) and multiple parasitosis in Formosa (69.2%). The most frequent species in the majority of provinces were *Blastocystis sp*. and *Enterobius vermicularis*. Misiones presented the highest frequency of soil-transmitted helminthes (23.3%) and Mendoza the lowest (0.6%); none were found in Chubut, La Pampa, or Salta. Buenos Aires, Formosa, and Misiones presented a similar species composition, as did Chubut and La Pampa.

**Conclusions.:**

The frequency of parasitosis in Argentina corresponds to the country’s complex mosaic of climatic and socioeconomic variability and shows a declining trend from north to south and from east to west.

Las enfermedades parasitarias intestinales continúan siendo uno de los principales problemas de salud pública en el mundo por el gran número de personas afectadas. Según datos publicados por la Organización Mundial de la Salud en 2001, aproximadamente 3 500 millones de personas sufrían de parasitosis y enfermedades asociadas (1). Asimismo, se estima que alrededor de 2 000 millones de personas pueden estar infestadas por al menos una especie de geohelmintos (como *Ascaris lumbricoides*, anquilostomas, *Trichuris trichiura*) y 4 000 millones se encuentran en riesgo de infestación (2). Estas parasitosis son más frecuentes en países en desarrollo, en constante crecimiento demográfico y con inadecuada infraestructura sanitaria y ambiental (3).

Algunos autores han sugerido que las enteroparasitosis están relacionadas, por una parte, con factores geográficos y socioeconómicos, y por otra con el estado nutricional de los niños, debido a que conducen al retraso del crecimiento por inapetencia, competencia por los nutrientes, anemia por deficiencia de hierro, diarrea y síndrome de malabsorción, entre otros trastornos (4, 5). Los casos de poliparasitosis que causan infestaciones crónicas agravan aun más el cuadro clínico, especialmente en niños de edad escolar (6, 7). A pesar de los avances de los últimos años en el conocimiento epidemiológico y el desarrollo de nuevas estrategias para el control, la frecuencia de estas infestaciones se ha mantenido estable debido, entre otras causas, a que las tasas de mortalidad son inferiores a las de otras enfermedades (8).

En América Latina, estudios epidemiológicos han mostrado prevalencias parasitarias que varían entre 30% y 53% (9–11). En Argentina, tanto la prevalencia de enteroparasitosis como el espectro de especies predominantes varían considerablemente de una localidad a otra (12, 13); se han registrado prevalencias por encima de 80% en el norte y el sur del país (14-18), mientras que en la zona central los valores se sitúan entre 40% y 70% (19-21).

El territorio de la República Argentina está constituido por un mosaico de eco-regiones con características climáticas, geomorfológicas y biológicas contrastantes (22). Estas diferencias regionales se manifiestan también en lo económico y lo social. Al respecto, Porto (23) realizó la caracterización de las provincias argentinas a partir de indicadores relacionados con variables sociales (entre ellas, la densidad poblacional, la tasa de analfabetismo, la calidad educativa, la tasa de mortalidad infantil, el hacinamiento en el hogar, el tipo de vivienda y las condiciones sanitarias) y económicas (como el consumo de energía eléctrica per cápita, las tasas de incidencia de pobreza, de actividad, y de empleo y desempleo). Estos indicadores permitieron clasificar las provincias según su condición socioeconómica en cuatro grupos, definidos previamente por Núñez Miñana (24): avanzadas (Santa Cruz, Chubut, La Pampa y Neuquén), especiales (Buenos Aires, Santa Fe, Córdoba y Río Negro), intermedias (Mendoza, Entre Ríos, San Luis, Catamarca, San Juan y Tucumán) y rezagadas (La Rioja, Salta, Jujuy, Misiones, Corrientes, Santiago del Estero, Chaco y Formosa).

En este contexto, el objetivo del presente trabajo fue determinar la distribución de las enteroparasitosis en niños de nueve provincias representativas del mosaico de ambientes contrastantes de Argentina.

## MATERIALES Y MÉTODOS

En este estudio descriptivo, observacional y transversal se incluyeron niños de uno u otro sexo con edades entre 0 y 14 años, los cuales se agruparon en dos intervalos de edad: de 5 años o menos (preescolares) y de 6 a 14 años (escolares).

Participaron en el estudio los niños y niñas que asistían a los establecimientos educativos y sus hermanos, que aceptaron participar de manera voluntaria y contaban con el consentimiento informado de sus padres o tutores. Se excluyeron los que hubieran recibido tratamiento antiparasitario al momento del relevamiento.

Debido a las diferencias en la densidad poblacional de las localidades estudiadas y a que la participación de la población fue estrictamente voluntaria, para determinar la representatividad de la muestra analizada se utilizó la curva de acumulación de especies parásitas, según el programa estadístico EstimateS (25).

### Área de estudio

**FIGURA 1.**
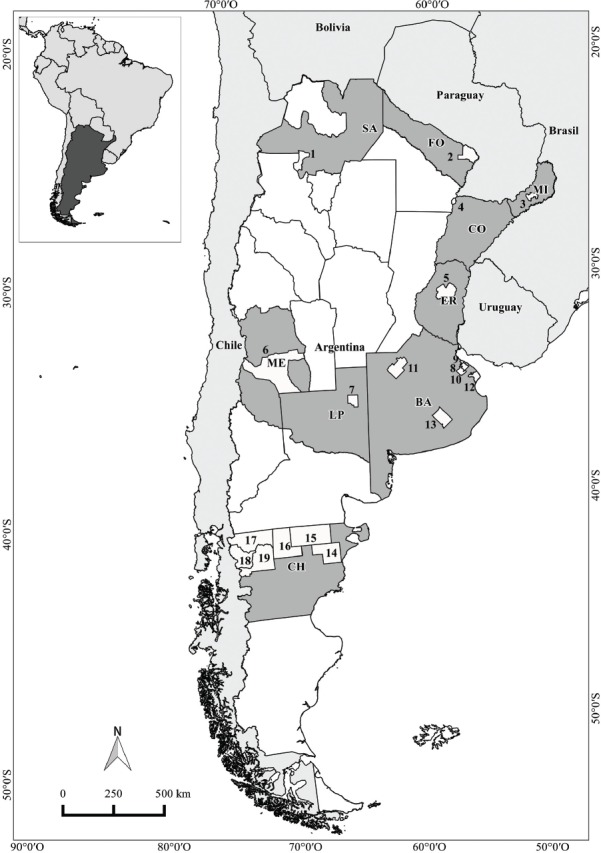
Ubicación geográfica de la República Argentina y las poblaciones estudiadas

La República Argentina se encuentra ubicada en el extremo meridional de América del Sur; limita al norte con Bolivia y Paraguay, al nordeste con Brasil, al este con Uruguay y el océano Atlántico, y al oeste con Chile. El estudio abarcó las siguientes regiones geográficas, departamentos y provincias (figura 1):

#### 1. Norte.

El departamento Molinos, en la provincia de Salta, presenta un clima subtropical seco y frío, con una marcada variación térmica diaria. Las lluvias son escasas, menores de 200 mm al año; su aridez limita la evolución de los suelos, que son pedregosos, pobres en materia orgánica, salinos y con escasa vegetación.

El departamento Pilcomayo, en la provincia de Formosa, presenta un clima subtropical, con una muy marcada variación térmica estacional. Hacia el este, las lluvias son uniformes durante todo el año y pueden ser superiores a 1200 mm anuales. Los suelos son arcillo-limosos, con drenaje pobre a imperfecto.

Los departamentos Capital, en la provincia de Corrientes; Cainguás, en Misiones; y Villaguay, en Entre Ríos, presentan clima tropical-subtropical templado, sin estación seca. En Misiones, los suelos, ricos en óxidos de hierro y aluminio, suelen ser profundos, con drenaje bueno a moderado y con amplia cobertura vegetal. En Entre Ríos predominan suelos fuertemente arcillosos y mal drenados, mientras que en Corrientes, la concentración estacional de las lluvias y la escasa pendiente dan lugar al desborde de los ríos y a frecuentes inundaciones (22).

#### 2. Centro.

El departamento San Rafael, en la provincia de Mendoza, presenta un clima templado seco, con suelos semiáridos, una temperatura media anual de 15 °C y precipitaciones medias anuales cercanas a 300 mm.

El departamento Capital, en la provincia de La Pampa, posee un clima templado, con una estación seca invernal; su temperatura media anual es de 13 °C y el promedio anual de precipitaciones es de 610 mm. Predominan suelos drenados de texturas excesivamente arenosas.

Los departamentos La Plata, Berazategui, Brandsen, Lincoln, Punta Indio y Tandil, en la provincia de Buenos Aires, presentan, en general, un clima templado húmedo con temperaturas medias anuales entre 15 °C y 18 °C; las lluvias, distribuidas durante el año, varían entre 600 y 1 100 mm. Los suelos, en general, son ricos en nutrientes y materia orgánica (22).

#### 3. Sur.

Los departamentos Gaiman, Telsen, Gastre, Cushamen, Futaleufú y Languiñeo, todos en la provincia de Chubut, presentan un clima de templado árido a templado frío húmedo; en el centro de la provincia el clima es frío y seco. Las temperaturas medias anuales varían entre 5 °C y 14 °C y las precipitaciones son menores de 250 mm. Los suelos predominantes son de textura gruesa, pedregosa, ricos en carbonato de calcio y pobres en materia orgánica (22).

***Fuente:*** elaborado por los autores.

Leyenda

Departamentos

1: Molinos, 2: Pilcomayo, 3: Cainguás, 4: Corrientes Capital, 5: Villaguay, 6: San Rafael, 7: La Pampa Capital, 8: La Plata, 9: Berazategui, 10: Brandsen, 11: Lincoln, 12: Punta Indio, 13: Tandil, 14: Gaiman, 15: Telsen, 16: Gastre, 17: Cushamen, 18: Futaleufú, 19: Languiñeo

### Relevamiento coproparasitológico

Los padres o tutores colectaron por la mañana durante 5-7 días consecutivos las muestras de materia fecal y los escobillados de la zona perianal con gasa estéril, en frascos con formol al 10%, según las instrucciones verbales y escritas provistas por el equipo de trabajo. Cada niño se evaluó una sola vez.

Las muestras se analizaron en el Laboratorio de Biodiversidad y Epidemiología Parasitaria del Centro de Estudios Parasitológicos y de Vectores, La Plata, Buenos Aires, Argentina. Para las muestras fecales se utilizó la técnica de Ritchie con un segundo lavado para obtener un sobrenadante límpido. Los escobillados anales conservados en formol se agitaron vigorosamente y se centrifugaron a 400 g durante 10 minutos para obtener un sedimento con la mayor concentración posible de huevos de *Enterobius vermicularis* (26).

Las muestras correspondieron a relevamientos llevados a cabo por diferentes planes de investigación y financiamiento entre 2005 y 2014, en dependencia de la provincia: Buenos Aires, entre 2005 y 2013; Chubut, entre 2010 y 2013; Corrientes, en 2012; Entre Ríos, entre 2010 y 2012; Formosa, en 2014; La Pampa, en 2006; Mendoza, entre 2008 y 2011; Misiones, entre 2005 y 2008 y en 2013; y Salta, entre 2012 y 2013.

### Análisis estadístico

Para el análisis de los datos obtenidos se empleó el paquete informático Epi Info 7. Se calcularon la riqueza específica (S: número de especies parásitas encontradas) y la frecuencia de parasitosis, así como el porcentaje de infectados por una sola especie (monoparasitosis) y por dos o más especies (parasitosis múltiple). Además, se calculó el coeficiente de similitud de Sørensen, que expresa en términos porcentuales el grado de similitud entre provincias, según las especies de parásitos encontradas (27).

Las asociaciones estadísticas se establecieron mediante la prueba de la *χ*^2^ con la corrección de Yates y la prueba exacta de Fisher (para menos de 5 observaciones). El nivel de significación fue de *p* < 0,05 o *p* < 0,01, según el caso.

### Aspectos éticos

El trabajo se desarrolló sin afectar a la integridad física, psíquica y moral de los participantes, con resguardo de su identidad y el consentimiento por escrito firmado por los padres o tutores. El presente trabajo recibió la aprobación del Comité de Ética de la Escuela Latinoamericana de Bioética (CELABE), bajo la resolución N.º 003/2016 Acta 73. El estudio se ajustó a lo establecido en la Declaración Universal de los Derechos Humanos de 1948, las normas éticas instituidas por el Código de Núremberg de 1947 y la Declaración de Helsinki de 1964 y sus sucesivas enmiendas, atendiéndose especialmente a lo normado por el artículo 5° del Decreto Reglamentario de la Ley Nacional 25326.

## RESULTADOS

En total, se analizaron 3 626 niños (49,6% varones y 50,4% mujeres). De ellos, 1 111 eran de la provincia de Buenos Aires, 203 de Chubut, 60 de Corrientes, 268 de Entre Ríos, 93 de Formosa, 58 de La Pampa, 752 de Mendoza, 1 015 de Misiones y 66 de Salta. Los niños de 5 años o menos constituyeron el 46,9% del total y el menor tenía 6 meses de edad.

Según el porcentaje total de niños parasitados por provincia, la mayor frecuencia se observó en Misiones y la menor en Chubut (*χ*^2^ = 167,5; *p* < 0,01). Con respecto al sexo, las diferencias no fueron significativas en las provincias estudiadas, excepto en Buenos Aires, donde los varones estaban más parasitados que las mujeres (*χ*^2^ = 3,8; *p* < 0,05). Por intervalos de edad, los más parasitados resultaron los preescolares de Buenos Aires (*χ*^2^ = 10,7; *p* < 0,01) y los escolares de Mendoza (*χ*^2^ = 5,8; *p* < 0,05) y Misiones (*χ*^2^ = 13,9; *p* < 0,01) (cuadro 1).

En relación con la riqueza específica, Misiones y Buenos Aires presentaron el mayor número de especies (S = 12), seguidos de Formosa y Mendoza (S = 10), Entre Ríos (S = 9), Corrientes (S = 8), Salta (S = 7), y Chubut y La Pampa (S = 6).

El porcentaje de monoparasitosis fluctuó entre 30,8% y 67,9%, más frecuente en las provincias de Chubut, Corrientes y Entre Ríos. La frecuencia de parasitosis múltiples osciló entre 32,1% y 69,2%, con los valores más altos en Formosa, Misiones y Buenos Aires. Las provincias de La Pampa, Mendoza y Salta mostraron valores intermedios para ambas formas de parasitosis.

Sobre el total analizado en cada provincia, la frecuencia de protozoos patógenos varió entre 19,2% en Chubut y 66,5% en Misiones. En el caso de los protozoos comensales (no patógenos), se observaron valores más bajos que fluctuaron entre 8,3% en Corrientes y 30,1% en Formosa. Misiones presentó la frecuencia de geohelmintos más elevada (23,3%) y Mendoza, la más baja (0,6%); no se hallaron geohelmintos en Chubut, La Pampa y Salta. Las frecuencias de otros helmintos (*Hymenolepis nana* y *E. vermicularis*) oscilaron entre 13,6% en Salta y 48,3% en La Pampa. Las diferencias observadas entre las frecuencias totales de protozoos y helmintos fueron significativas en todas las provincias (cuadro 2).

**CUADRO 1.** Frecuencias absoluta y relativa (%) de niños parasitados en las provincias argentinas analizadas, por sexo e intervalo de edad

Variable	Casos detectados (%)								
	Buenos Aires	Chubut	Corrientes	Entre Ríos	Formosa	La Pampa	Mendoza	Misiones	Salta
Sexo^a^									
Varones	390 (52,6)	44 (56,4)	15 (51,7)	82 (52,2)	36 (54,5)	20 (51,3)	231 (49,6)	396 (47,6)	17 (42,5)
Mujeres	351 (47,4)	34 (43,6)	14 (48,3)	75 (47,8)	30 (45,5)	19 (48,7)	235 (50,4)	436 (52,4)	23 (57,5)
Intervalo de edad^b^									
5 años o menos	462 (62,3)	55 (70,5)	20 (69,0)	96 (61,2)	6 (9,1)	36 (92,3)	152 (32,6)	165 (19,8)	29 (72,5)
6-14 años	279 (37,7)	23 (29,5)	9 (31,0)	61 (38,8)	60 (90,9)	3 (7,7)	314 (67,4)	667 (80,2)	11 (27,5)
Total^c^	741 (66,7)	78 (38,4)	29 (48,3)	157 (58,6)	66 (69,9)	39 (65,5)	466 (62,0)	832 (82,0)	40 (60,7)

***Fuente:*** elaborado por los autores a partir de los resultados del estudio.

a Diferencia significativa entre las frecuencias de niños parasitados por sexo en Buenos Aires (*p* < 0,05).

b Diferencia significativa entre las frecuencias de niños parasitados por intervalo de edad en Buenos Aires, Mendoza y Misiones (*p* < 0,05).

c Diferencia significativa entre las frecuencias de niños parasitados en Chubut y Misiones (*p* < 0,01).

La frecuencia de infestación con cada especie se muestra en el cuadro 3. Entre los protozoos comensales, *Entamoeba coli* fue más frecuente en la mayoría de las provincias, con excepción de Corrientes y Salta, donde fue *Endolimax nana*. El patógeno más frecuentemente detectado en todas las provincias fue *Blastocystis* sp., excepto en Corrientes donde fue *Giardia lamblia*. En el caso de los helmintos, *E. vermicularis* fue el más frecuentemente encontrado en todas las provincias; se observó una elevada frecuencia de anquilostomideos en Misiones.

Con respecto a los anquilostomideos en la provincia de Misiones, los varones resultaron más frecuentemente parasitados que las mujeres (57,3% *vs*. 42,7%; *χ*^2^ = 8,5; *p* < 0,01) y los escolares más que los preescolares (89,1% *vs*. 10,9%; *χ*^2^ = 13,4; *p* < 0,01), al igual que con *Strongyloides stercoralis* (87,6% *vs*. 12,4%; *χ*^2^ = 6,4; *p* < 0,01).

Entre las asociaciones más frecuentes y significativas (*p* < 0,05) se destacan las encontradas entre *E. coli* y *E. nana; E. coli* y *Blastocystis* sp.; *E. coli* y *G. lamblia*; y *Blastocystis* sp. y *G. lamblia* en Buenos Aires y Misiones. La asociación entre *Blastocystis* sp. y *G. lamblia* se halló también en Chubut, Corrientes, Entre Ríos y Mendoza. Con respecto a los helmintos, se encontró asociación entre la presencia de *E. vermicularis* y *H. nana*, con *E. coli, Blastocystis* sp. y *G. lamblia* en Buenos Aires, Corrientes, La Pampa, Mendoza y Misiones.

**CUADRO 2.** Frecuencia de protozoos y helmintos en las provincias argentinas analizadas

Provincia	Protozoos		Helmintos	
	Patógenos	Comensales	Geohelmintos	No geohelmintos
Buenos Aires^b^	42,2	23,8	5,7	38,8
Chubut^b^	19,2	11,3	0,0	21,2
Corrientes^b^	26,7	8,3	6,7	25,0
Entre Ríos^b^	33,2	8,9	0,7	38,8
Formosa^b^	61,3	30,1	7,5	30,1
La Pampa^b^	41,4	8,6	0,0	48,3
Mendoza^b^	48,9	17,0	0,6	25,3
Misiones^b^	66,5	20,1	23,3	43,6
Salta^b^	53,0	21,2	0,0	13,6

***Fuente:*** elaborado por los autores a partir de los resultados del estudio.

a Porcentaje calculado a partir del total de niños y niñas analizados por provincia.

b Diferencia significativa entre las frecuencias totales de protozoos y de helmintos, *p* < 0,05.

Entre los geohelmintos, se observó la asociación entre la presencia de *A. lumbricoides* y *T. trichiura* en Buenos Aires, así como de *S. stercoralis* y *A. lumbricoides,* y *S. stercoralis* y anquilostomideos en Misiones (*p* < 0,01).

Según el coeficiente de similitud de Sørensen, la composición de especies parasitarias fue similar en Buenos Aires, Formosa, Mendoza y Misiones, con valores superiores a 90,0%. También se observó un valor muy elevado (91,0%) entre Chubut y La Pampa. En el otro extremo, estas últimas provincias, junto con Salta, mostraron valores de similitud iguales o inferiores a 66,0% con respecto a Corrientes, Formosa, Mendoza y Misiones (cuadro 4).

## DISCUSIÓN

En los últimos años, la globalización y las migraciones humanas desde regiones endémicas han favorecido la dispersión de ciertas parasitosis, fenómeno potenciado por determinadas condiciones ambientales y económicas y las deficientes prácticas sanitarias (12). En consecuencia, algunas especies parasitarias muestran una distribución cosmopolita, mientras que otras mantienen una distribución geográfica específica. Argentina presenta una gran diversidad de suelos y condiciones climáticas y, en este escenario, es posible hallar parásitos que requieren condiciones muy diversas para su transmisión (12). Esto se confirma con los resultados del presente trabajo, que muestran la heterogeneidad en la distribución de las enteroparasitosis en las poblaciones infantiles aquí estudiadas, en correspondencia con las características de cada región.

**CUADRO 3.** Frecuencia de protozoos comensales, patógenos, y helmintos en las provincias argentinas analizadas

Especie	Provincia								
	Buenos Aires	Chubut	Corrientes	Entre Ríos	Formosa	La Pampa	Mendoza	Misiones	Salta
Protozoos comensales									
*Entamoeba coli*	14,2	6,9	3,3	5,6	20,4	6,9	14,6	14,9	7,6
*Endolimax nana*	10,5	3,5	5,0	4,5	12,9	1,7	2,7	6,5	15,2
*Enteromonas hominis*	5,5	1,5	0,0	0,4	0,0	0,0	0,0	0,6	0,0
*ChiIomastix mesnili*	1,4	0,0	0,0	0,0	1,1	0,0	0,5	0,5	3,0
*Iodamoeba bütschlii*	0,8	0,0	0,0	1,1	1,1	0,0	0,5	1,4	1,5
Protozoos patógenos									
*Giardia lamblia*	13,9	5,9	20,0	11,9	31,2	15,5	18,8	20,2	27,3
*Blastocystis* sp.	33,9	16,3	16,7	27,2	49,5	34,5	44,8	59,6	40,9
Helmintos									
*Hymenolepis nana*	3,2	0,0	5,0	1,1	10,8	1,7	1,1	5,1	0,0
*Enterobius vermicularis*	37,7	21,7	20,0	39,2	20,4	50,9	24,8	42,9	13,6
*Ascaris lumbricoides*	5,2	0,0	5,0	0,0	6,5	0,0	0,3	2,6	0,0
Anquilostomideos	0,0	0,0	0,0	0,0	0,0	0,0	0,4	16,2	0,0
*Trichuris trichiura*	1,9	0,0	1,7	0,8	0,0	0,0	0,0	0,0	0,0
*Strongyloides stercoralis*	0,2	0,0	0,0	0,0	1,1	0,0	0,0	11,1	0,0

***Fuente:*** elaborado por los autores a partir de los resultados del estudio.

a Porcentaje calculado a partir del total de niños y niñas analizados por provincia.

**CUADRO 4.** Coeficiente de similitud de Sørensen (%) según los casos de parasitosis encontrados en las provincias argentinas analizadas

Provincia	Buenos Aires	Chubut	Corrientes	Entre Ríos	Formosa	La Pampa	Mendoza	Misiones	Salta
Buenos Aires	100	88	80	85	91	88	91	92	77
Chubut		100	71	80	63	91	62	66	77
Corrientes			100	82	80	86	77	70	66
Entre Ríos				100	74	80	74	76	75
Formosa					100	80	90	91	80
La Pampa						100	75	66	77
Mendoza							100	91	82
Misiones								100	74
Salta									100

***Fuente:*** elaborado por los autores a partir de los resultados del estudio.

La mayor frecuencia de parasitosis en Misiones (nordeste) contrasta con la encontrada en Chubut (sur), lo que coincide en parte con lo informado para otras localidades del norte (Corrientes, Salta y Tucumán) (14, 28, 29) y el sur del país (Neuquén) (30). Evidentemente, las condiciones climáticas y los suelos de Misiones favorecen la presencia y la transmisión de enteroparásitos, algo que el clima frío y seco y los suelos pobres en materia orgánica de Chubut no favorecen. Además de estos factores limitantes del desarrollo de estos parásitos, las particularidades socioeconómicas de Misiones (clasificada como una provincia rezagada) y Chubut (provincia avanzada) contribuyen a estas diferencias.

Las parasitosis múltiples pueden afectar más al estado de salud de las personas infestadas, especialmente cuando involucran varias especies patógenas (7). En este sentido, el número de casos de infestación múltiple y de especies halladas fue mayor en Misiones y Formosa (provincias rezagadas) y menor en Chubut (provincia avanzada).

En general, el porcentaje de niños parasitados en la población estudiada no mostró diferencias según el sexo, con la excepción de Buenos Aires, donde se observó mayor frecuencia en los varones. Sin embargo, esto podría estar determinado más por aspectos culturales y conductuales específicos de la población estudiada que por el sexo en sí.

En cuanto a la edad, los niños (varones y mujeres) de 5 años o menos de Buenos Aires y los de 6 años o más de Misiones y Mendoza resultaron estar más parasitados. Diferentes investigaciones han mostrado resultados disímiles en relación al sexo y la edad de los individuos, sin llegar a un consenso (31, 32). Sin embargo, se ha sugerido que la parasitosis intestinal en escolares tiene su origen en la relación que ellos establecen con las fuentes de infestación y depende de las prácticas de juego y los hábitos de higiene (33, 34).

Entre las especies halladas, *Blastocystis* sp., *E. vermicularis* y *G. lamblia* fueron las más frecuentes en la mayoría de las provincias, en concordancia con otras investigaciones (14, 32, 35). Entre las provincias estudiadas, la mayor frecuencia de *Blastocystis* sp. correspondió a Misiones y la menor a Chubut. La infestación por este parásito no parece explicarse por las diferencias socioeconómicas, las condiciones climáticas o las áreas geográficas. Su papel como patógeno aún no está claro y se desconoce si es puramente comensal o se comporta como patógeno bajo determinadas circunstancias, por ejemplo, cuando coexiste con otros parásitos o si la carga parasitaria es elevada (9). A pesar de que se ha esclarecido su ubicación taxonómica, identificación de subtipos y carácter zoonótico (36, 37), aún permanecen sin dilucidar aspectos de su biología y epidemiología.

*Giardia lamblia* mostró en Formosa la frecuencia más alta y en Chubut la más baja. La giardiasis, principal causa de diarrea no viral ni bacteriana, afecta a millones de personas en todo el mundo (21, 36); es frecuente en climas cálidos y templados y varios autores han informado de su presencia en Argentina (20, 28, 30). Al igual que *Blastocystis* sp. y otros protozoos, la infección por *G. lamblia* puede ocurrir por el consumo de agua o alimentos no seguros o el uso de objetos contaminados con quistes (36, 38). Generalmente, se asocia con otras especies comensales indicadoras de contaminación fecal (por ejemplo, *E. nana, E. coli y E. hominis*) o la falta de servicios hidrosanitarios (hogares sin cloaca o agua corriente) (37, 39), algo frecuente en las poblaciones analizadas y un escenario epidemiológico habitual en diferentes localidades argentinas (39).

En cuanto a *E. vermicularis,* se encontró una elevada frecuencia en la mayoría de las provincias estudiadas. Este parásito cosmopolita —considerado más una molestia que una causa de enfermedad grave— ocasiona una elevada morbilidad caracterizada por prurito anal, irritabilidad, somnolencia y falta de concentración. Afecta principalmente a niños y niñas en edad escolar que aún no incorporaron hábitos higiénicos adecuados y en los que es frecuente la onicofagia y el deficiente lavado de las uñas y las manos, lo que favorece su transmisión. El hacinamiento y el compartir camas y ropa constituyen otros factores que predisponen a esta infestación (40, 41).

Con respecto a los geohelmintos, Misiones presentó la máxima frecuencia y Mendoza la menor (23,3% frente a 0,6%, respectivamente), en tanto no se hallaron en Chubut, La Pampa y Salta. Estudios previos realizados en Misiones mostraron prevalencias mayores de 60% en comunidades aborígenes y de 20% en la población conformada por migrantes y nativos no aborígenes (42). Estas infestaciones son endémicas en áreas tropicales y subtropicales, principalmente en países en desarrollo, y constituyen un indicador de las condiciones sanitarias y ecológicas del entorno de sus hospederos (43, 44). Los valores de humedad relativa y temperatura ambiental, así como el tipo de suelo en el cual se desarrollan los huevos y las larvas, determinan la distribución de estas especies (13). Así, las elevadas frecuencias de anquilostomideos y *S. stercoralis* observadas en Misiones se explicarían por su clima cálido, la elevada humedad relativa y la abundante cobertura vegetal, sumada al hábito de defecar a cielo abierto, la falta de una correcta eliminación de excretas y el deficiente abastecimiento de agua potable (6, 42). En contraste, la ausencia de geohelmintos en Chubut, La Pampa y Salta puede deberse al clima imperante en esas provincias —ya sea templado o frío seco—, a la alta radiación solar, y al suelo pobre en materia orgánica y fuertemente erosionado, que caracterizan a Chubut y los Valles Calchaquíes de Salta. Soriano y colaboradores (30) no observaron geohelmintos en una población de Neuquén (noroeste de la Patagonia argentina), donde el clima frío y la fitogeografía son semejantes a los de Chubut. Por el contrario, Taranto y colaboradores (16) encontraron en Tartagal, al noreste de Salta, altas prevalencias de *S. stercoralis* y anquilostomideos en comunidades aborígenes provenientes de zonas selváticas de Bolivia, que poseen características edafológicas y climáticas diferentes a las áreas estudiadas en el presente estudio.

Por otra parte, los pocos casos de *S. stercoralis* encontrados en Buenos Aires y de anquilostomideos en Mendoza pueden atribuirse a las migraciones de personas desde zonas endémicas (19). En otros estudios se encontraron pocos casos de anquilostomideos en Buenos Aires (20, 45).

En cuanto a *A. lumbricoides,* fue más frecuente en Formosa y estuvo ausente en Entre Ríos, mientras que *T. trichiura* resultó más frecuente en Buenos Aires y no se observó en Mendoza, Formosa y Misiones. La ausencia o baja frecuencia de estas especies podría explicarse por tratarse de lugares sometidos a frecuentes inundaciones (principalmente Misiones) como resultado de la considerable dinámica fluvial que presentan estas áreas, lo que provocaría la infiltración de los huevos hacia mayores profundidades (46). Burden y colaboradores (47) indicaron que la presencia de huevos a 60 cm de profundidad disminuye sus posibilidades de entrar en contacto con el hospedero en la superficie y, por lo tanto, de infestarlo. Además, se sabe que tanto la radiación solar como la humedad excesiva sobre el suelo producen la inviabilidad del huevo (48).

La asociación observada entre diferentes geohelmintos, en particular en Buenos Aires y Misiones, se ve reforzada por factores ambientales favorables, las deficiencias en el saneamiento ambiental y algunos factores socioculturales, como la defecación a cielo abierto, el andar descalzo y el contacto estrecho con el suelo (44–46).

En este estudio se halló una gran variedad de combinaciones de protozoos patógenos y comensales, probablemente porque comparten la misma vía de transmisión y su presencia se ve favorecida por factores higiénico-sanitarios deficientes, entre ellos el consumo de agua y alimentos contaminados con materia fecal, las características de los suelos y la insuficiente higiene personal (12, 31).

El coeficiente de similitud de Sørensen observado entre Buenos Aires, Formosa, Mendoza y Misiones indica una composición de especies parásitas semejante, principalmente de protozoos. En el otro extremo del espectro, Chubut y La Pampa se alejan de las anteriores por la ausencia de geohelmintos. Si bien se observó que Mendoza presentó similitud con Buenos Aires, Misiones y Formosa, eso podría responder a la presencia de *A. lumbricoides* y anquilostomideos, pero con muy baja frecuencia (< 1%).

El presente trabajo da cuenta de la distribución heterogénea de las enteroparasitosis en la población infantil residente en varias provincias representativas de las diferentes regiones de Argentina; sin embargo, se deben tener en cuenta algunas limitaciones para una correcta interpretación de los resultados. En primer lugar, los relevamientos coproparasitológicos se realizaron en diferentes años y no simultáneamente en todas las provincias; no obstante, por tratarse de un estudio descriptivo, transversal y no comparativo, esas diferencias temporales no deben interferir de manera significativa en estos resultados. Además, los estudios orientados a caracterizar las provincias argentinas desde el punto de vista económico y social han demostrado que en los últimos años la distribución espacial de la riqueza *vs*. pobreza y las condiciones sociales y ambientales asociadas no se han modificado sustancialmente.

Se observó que la población infantil residente en las provincias rezagadas desde el punto de vista socioeconómico y con características ambientales favorables para el desarrollo y la infestación parasitaria, tales como Misiones y Formosa, presentaron mayor número de especies parásitas y porcentaje de infestados. En el otro extremo del país, la población estudiada de Chubut, una provincia socioeconómicamente avanzada y con características ambientales muy diferentes, mostró valores más bajos. Los porcentajes de parasitosis en las restantes provincias se ubicaron en una posición intermedia.

En resumen, las frecuencias de parasitosis en Argentina responden al complejo mosaico de variabilidad climática y socioeconómica del país y revelan una tendencia descendente de norte a sur y de este a oeste.

Se debe profundizar el conocimiento sobre las afecciones parasitarias en otras poblaciones humanas, a fin de establecer referencias regionales y llegar a una mejor interpretación de la situación ambiental, sociocultural y epidemiológica del país. Sin duda, ello facilitará el diseño y la implementación de programas de prevención adecuados a cada realidad local.

### Agradecimientos.

Los autores agradecen a las autoridades locales, a la comunidad educativa y a los pobladores de las provincias estudiadas por su colaboración. Este trabajo fue financiado con fondos provenientes de la Universidad Nacional de La Plata (UNLP-11/N 679), por la Agencia Nacional de Promoción Científica y Tecnológica (PICT 1541) y por el Consejo Nacional de Investigaciones Científicas y Técnicas (PIP 00734).

### Declaración.

Las opiniones expresadas en este manuscrito son responsabilidad del autor y no reflejan necesariamente los criterios ni la política de la *RPSP/PAJPH* y/o de la OPS.
